# A Rare Case of Sudden Fatal Airway Obstruction Caused by an Undiagnosed Laryngeal Lesion

**DOI:** 10.7759/cureus.72103

**Published:** 2024-10-22

**Authors:** Teodora Kiryakova, Iviana Yovchevska, Veselin Tihchev, Alexandar Alexandrov, Ivan I Tsranchev

**Affiliations:** 1 Department of Forensic Medicine, Medical University Sofia, Sofia, BGR; 2 Department of Forensic Medicine, Medical Institute of the Ministry of Internal Affairs, Sofia, BGR; 3 Department of Physiology and Pathophysiology, Medical University Sofia, Sofia, BGR; 4 Department of Pathology and Forensic Medicine, University Hospital “Queen Ioanna”, Sofia, BGR; 5 Department of Forensic Medicine and Deontology, Medical University Sofia, Sofia, BGR; 6 Department of Forensic medicine and Deontology, Medical University of Plovdiv, Plovdiv, BGR

**Keywords:** acute airway obstruction, angiomixoid laryngeal polyp, benign laryngeal lesion, forensic medicine, mechanical asphyxia, pathophysiology, sudden death, undiagnosed lesion

## Abstract

Mechanical asphyxia is a severe condition caused by a physical blockage that impedes breathing in the upper airways, trachea, and lungs. We present a case of a 39-year-old man who died suddenly at home while getting ready for work. He had previously experienced shortness of breath and a sore throat. Despite consulting a pulmonologist, who ruled out lung diseases, no other specialist evaluations were conducted. We performed a comprehensive forensic investigation, including collecting medical history and criminological data, a forensic autopsy, and subsequent toxicological and histological analyses. The autopsy revealed a benign laryngeal lesion that completely blocked the upper airways. The cause of death was determined to be acute obstruction, leading to mechanical asphyxia. This case highlights the importance of comprehensive and timely medical evaluations to prevent serious life-threatening complications.

## Introduction

Asphyxia can be defined as a pathological process characterized by a lack of oxygen or excess carbon dioxide in the human body. A single functional complex, which includes the lungs, the gas transport function of blood, the cardiovascular system, cellular and respiratory enzymes, and neurohumoral and hormonal factors, regulates the oxygen balance in the body [1,2]. When a physical obstruction prevents breathing in the upper respiratory tract, trachea, or lungs, a critical condition occurs called mechanical asphyxia. Depending on the location and method of obstruction, it can be classified as compression of the neck (hanging, ligature, manual strangulation), compression of the thorax (traumatic asphyxia), compression of the nose and mouth (smothering), and obstruction of the upper airways by foreign bodies, tumors (malignant, benign), and liquids (drowning) [1]. This may prevent the airflow to the alveoli partially or completely [2,3]. Even though benign laryngeal lesions commonly present with different types of voice disorders, they rarely cause fatal airway obstruction [4-8].

The case was presented at the 27th European Congress of Pathology (ECP 2015), 5-9 September 2015, Belgrade, Serbia as an abstract.

## Case presentation

We present a case of a 39-year-old man who suddenly collapsed and died at home while dressing for work. He was found lying on his bedroom floor in a supine position, with signs of cyanosis on the face and petechial hemorrhages on the skin of the upper part of the body (face, neck, and thorax). Next to the bed of the deceased, three different types of analgesic and anti-inflammatory medications treating inflammatory conditions of the throat were observed. Anamnestic data collected at the scene from his roommate revealed that he had been complaining of shortness of breath and sore throat for a couple of months. The police investigation discovered that the deceased did not visit his general practitioner. He referred himself to a pulmonologist, who after a chest X-ray examination, concluded that there were no signs of lung inflammation or other parenchymal pathology. Consultations with other specialists were not made. After the scene examination, the body was transported for a routine forensic autopsy to determine the manner, mechanism, and cause of the death. 

**Figure 1:**
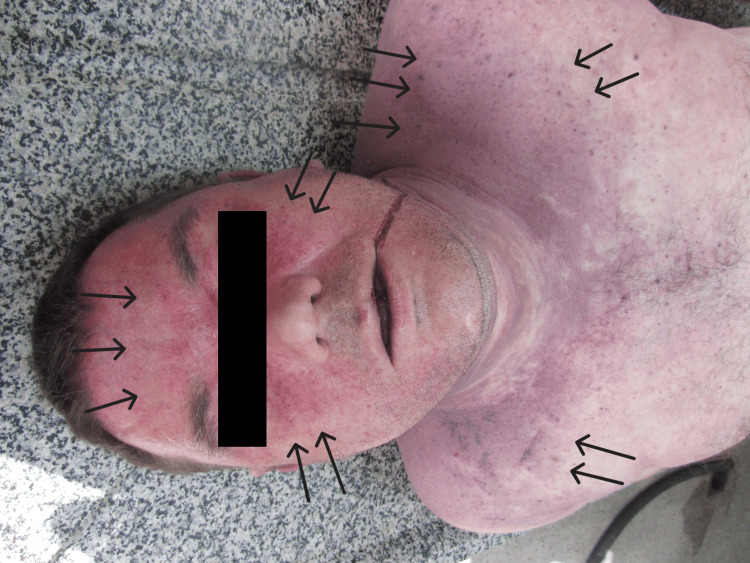
Vibices on the skin of the face, neck, and upper thorax

A full forensic autopsy of the deceased was performed with subsequent toxicological analysis and histological examination. He had a body weight of 97 kg and a height of 190 sm. During the external examination, we observed pronounced cyanosis on the face, neck, and upper third of the chest along with many punctate and diffuse hemorrhages (vibices) on the skin (Figure 1).

**Figure 2:**
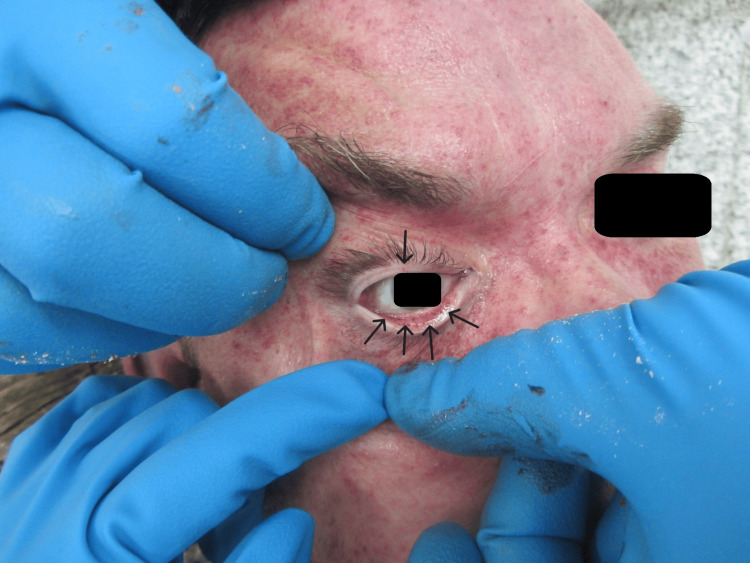
Petechial hemorrhages of the eyelids

Similar hemorrhages were seen on the mucous membrane of the eyelids- Tardieu spots (Figure 2). In the left temporal area, a pale bluish bruise was found. Other signs of traumatic injuries were not observed.

**Figure 3:**
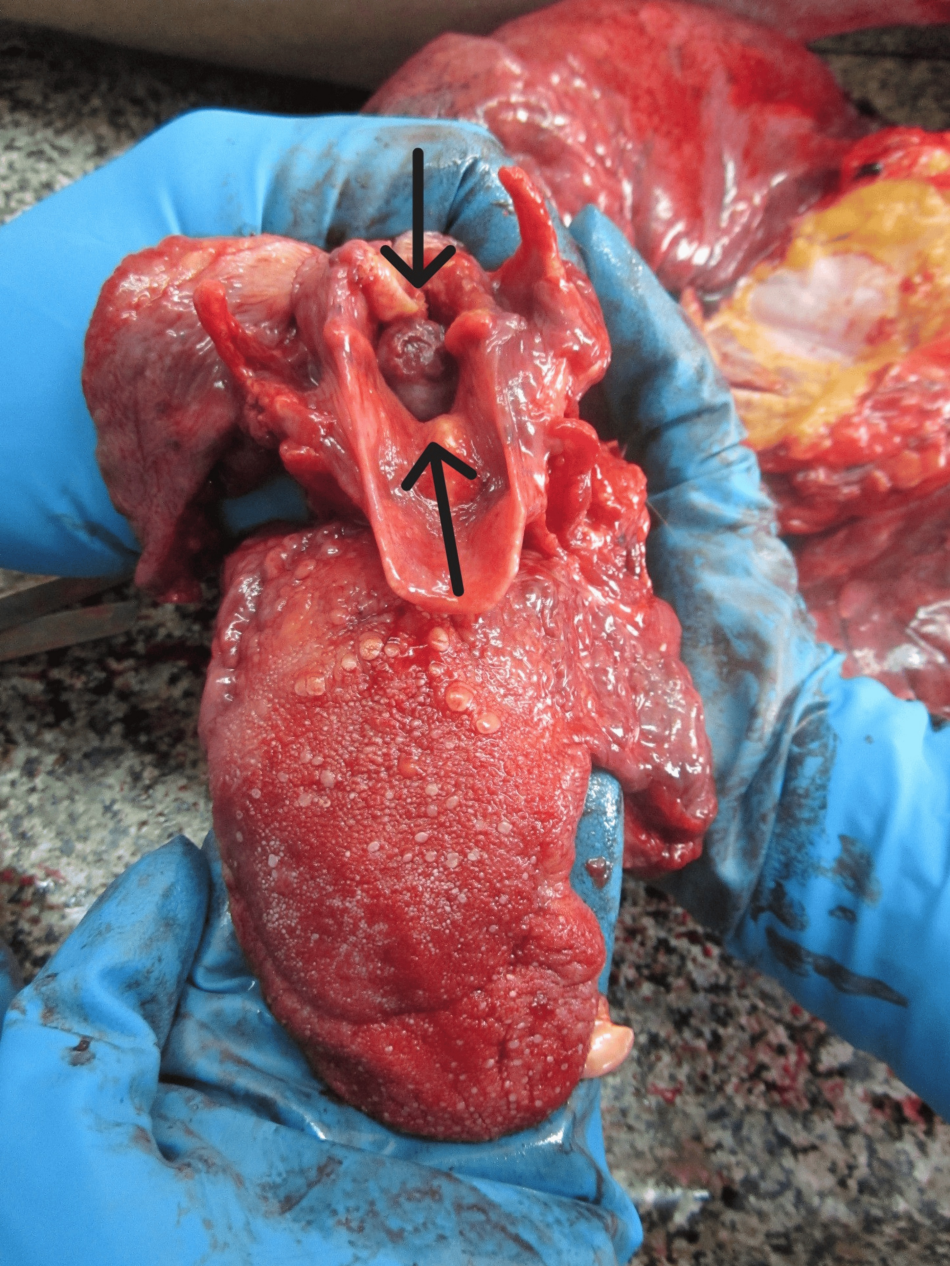
Laryngeal lesion, blocking the larynx

**Figure 4:**
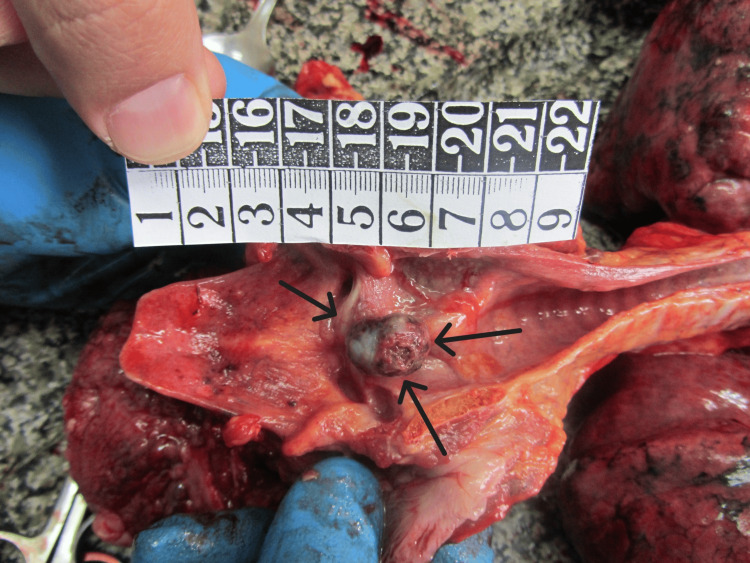
The laryngeal tumor, attached to the vocal cord

During the internal examination of the body at the entrance of the larynx, a large pedunculated tumor formation (1.8/1.4/0.5 cm) attached to the right vocal cord was observed (Figures 3, 4).

**Figure 5:**
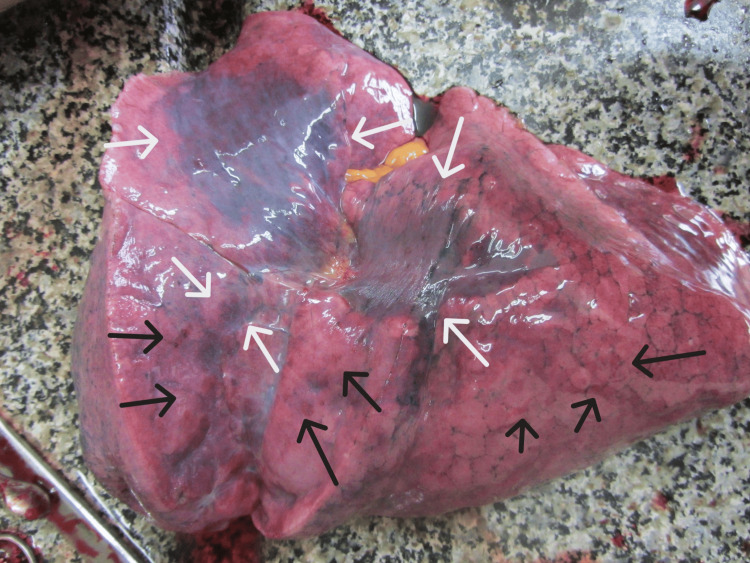
Left lung with petechial hemorrhages (black arrows) and atelectasis (white arrows)

**Figure 6:**
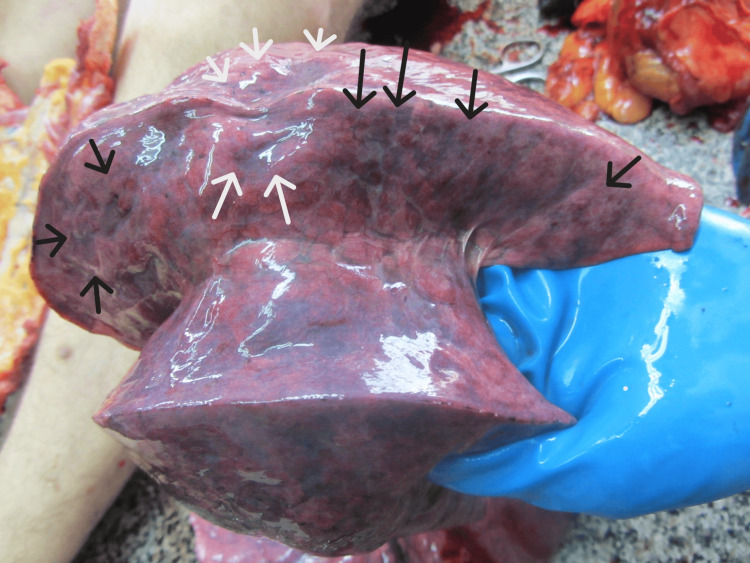
Right lung with petechial hemorrhages (black arrows) and atelectasis (white arrows)

It was completely obliterating the laryngeal aditus. The surrounding tissues were inflamed and edematous. The brain presented with severe congestion, edema, and a pronounced tonsillar herniation (weight 1,390 grams). The pleura was smooth and transparent, with scattered reddish punctate hemorrhages also observed on the epicardium. The lungs were congested and edematous (weighing 690 grams for the right and 650 for the left), with many petechial hemorrhages over the surface and areas of atelectasis (Figures 5, 6).

**Figure 7:**
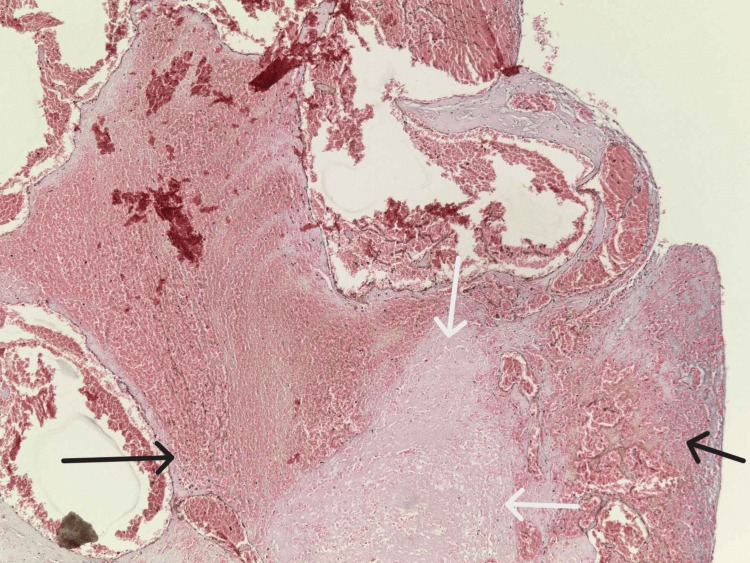
General histological image of the laryngeal polyp (HE, x10): myxoid edematous stromal changes, stromal hyalinization (white arrows), and telangiectatic blood vessels (black arrows)

**Figure 8:**
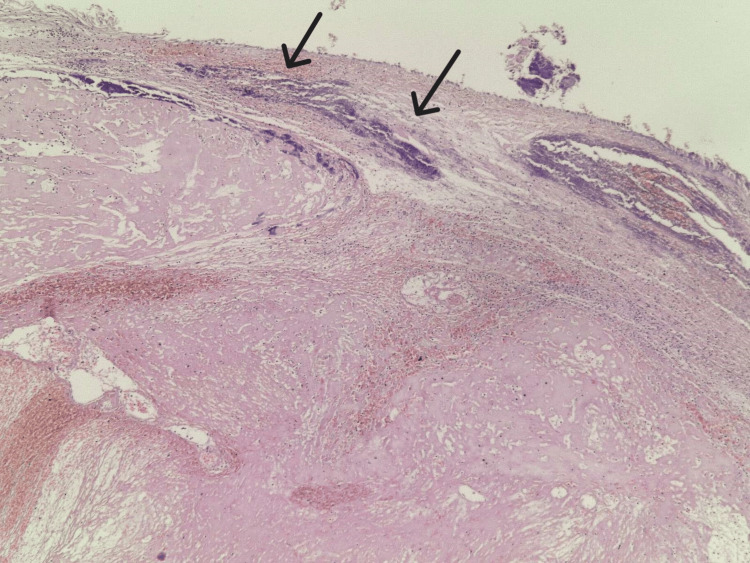
Laryngeal angiomyxoid polyp with bacterial colonies (HE, x40)

The heart was normal in size and weighed 330 grams with mild atherosclerotic changes of the coronary arteries. The blood was dark red and liquid. In addition, the abdominal organs presented with congestion; the liver weighed 1,610 grams, the spleen 240 grams, and the kidneys 125 grams each. Organ samples were preserved for histological examination. Well-presented brain and lung edema were observed with stasis in the internal organs. The microscopic examination of the tissues corresponded to the cause of death - asphyxia. The toxicological analysis was negative; the examined blood sample showed no ethyl alcohol or drug concentrations (alkaloids, synthetic and opiate narcotics, barbiturates, β-blockers, salicylates, phenothiazine, benzodiazepines, and imipramine drugs). The histological examination of the laryngeal lesion revealed an angiomyxoid laryngeal polyp (Figure 7) with myxoid edematous stromal changes, fibroblastic proliferation, stromal hyalinization, telangiectatic blood vessels, and squamous mucosa with ulceration and bacterial colonies on its surface (Figure 8).

Death was attributed to mechanical asphyxia due to acute obstruction of the airways caused by the laryngeal angiomyxoid polyp.

## Discussion

Any physical blockage of the airflow to the alveoli results in inadequate oxygen delivery to tissues, causing hypoxia (decreased oxygen levels in blood) and hypercapnia (accumulation of carbon dioxide in blood). Increased respiratory efforts follow but the physical obstruction prevents effective ventilation and the body loses its ability to compensate. As a result, the heart rate and blood pressure increase due to the autonomic nervous system [9]. However, prolonged oxygen deprivation eventually leads to progressive bradycardia and hypotension as a result of cardiac hypoxic injury. The subsequent cerebral hypoxia leads to neurological damage. The metabolic acidosis that develops due to the change from aerobic to anaerobic metabolism further impairs cellular and organ function [10,11]. In cases where the obstruction persists, systemic hypoxia causes multi-organ failure. Without timely intervention, this is followed by cardiac arrest and death [9,11].

Benign vocal cord lesions typically include singer’s nodules, papilloma, polyps, polypoid degenerations, and cysts [12]. These conditions are often linked to factors such as vocal trauma, voice misuse, or overuse (e.g., singing, screaming, cheerleading, excessive talking) [12,13]. Other potential causes are chronic upper airway infections accompanied by frequent coughing and throat-clearing [14], smoking, alcohol use, allergies [15], and in rare cases, hypothyroidism [16]. Pathological changes in vocal cord lesions occur primarily in the superficial layer of the lamina propria and the epithelium, where excessive mechanical trauma and stress lead to wound formation and remodeling [14]. Vocal cord polyps can be unilateral or bilateral, typically manifesting at the vocal cord's anterior and middle one-third junctions [12,17]. They come in various shapes and sizes, are generally more vascularized than nodules, and appear reddish in color. They are common in men between 30 and 50 years old, measuring up to several millimeters, and are attached to the free edges of the true vocal folds [18]. Respiratory issues caused by their presence are rare, and death due to airway obstruction from large laryngeal polyps is exceedingly uncommon [5-7].

The angiomyxoid laryngeal polyp is a unique subtype of benign laryngeal polyp characterized by a rare combination of vascular and myxoid tissue components. This distinct histological appearance, with blood vessels and a myxoid stroma, sets it apart from typical laryngeal polyps [16]. These polyps usually appear on the vocal cords or other areas of the larynx, often leading to vocal disturbances that can significantly affect voice quality. Comparable to other laryngeal polyps, angiomyxoid polyps may be linked to chronic larynx irritation, such as smoking, vocal overuse, or gastroesophageal reflux disease (GERD) [16]. The presence of the myxoid component in this variant may indicate a different or more unusual pattern of tissue response in certain individuals. However, the exact causes of angiomyxoid polyps are not fully understood.

In the presented case report, the deceased experienced symptoms that required medical attention. The deceased only consulted a pulmonologist. Unfortunately, for the sore throat and difficulty breathing that he had for a couple of months before his death, he didn’t visit his general practitioner. Consultation with an otorhinolaryngologist was not conducted as well. The laryngeal lesion remained undiagnosed. The lack of adequate medical consultation and evaluation resulted in a lethal outcome.

## Conclusions

Asphyxia may result from numerous etiologies and it is a common cause of sudden death. In general, vocal cord polyps are lesions that can cause dysphonia. In rare occurrences, they may be huge and present with dyspnea, choking, wheezing, and stridor. Obstruction from laryngeal polyps of the upper airways is rare. However, sudden airway obstruction may occur when a large pedunculated laryngeal polyp is unrecognized. Therefore, prompt diagnosis and treatment are important. Primary care physicians, endoscopists, anesthesiologists, and otolaryngologists should be aware of this condition and add it to their differential diagnosis of sudden respiratory obstruction. This case shows the importance of a complete and thorough analysis of every patient with proper, on-time diagnosis and treatment to avoid such complications. Examination of the oropharyngeal region by indirect laryngoscopy is an easy procedure. Clinicians should be well aware of the probability of vocal cord polyps and referral to an otolaryngologist should be made in all suspicious cases.
